# Evolving resistance patterns in *Tetranychus urticae* and *Bemisia tabaci* in Greece

**DOI:** 10.1002/ps.70475

**Published:** 2025-12-21

**Authors:** Anastasia Kampouraki, Aris Ilias, Kiriaki‐Maria Papapostolou, Styliani Malliaraki, Ioannis Pirgianakis, Evangelia Karakosta, John Vontas, Anastasia Tsagkarakou, Konstantinos Mavridis

**Affiliations:** ^1^ Institute of Molecular Biology and Biotechnology Foundation for Research and Technology Hellas (IMBB‐FORTH) Crete Greece; ^2^ Hellenic Agricultural Organization DIMITRA Institute of Olive Tree Subtropical Crops and Viticulture Heraklion Greece; ^3^ Pesticide Science Lab Agricultural University of Athens Athens Greece

**Keywords:** pesticide resistance, *Tetranychus urticae*, *Bemisia tabaci*, molecular diagnostics, green pesticides, integrated pest management (IPM)

## Abstract

**BACKGROUND:**

Pesticide resistance in agricultural pests remains a major barrier to effective and sustainable crop protection. In this study, we assessed the current resistance status of two key pests, *Tetranychus urticae* and *Bemisia tabaci*, in major agricultural regions of Greece. A total of 25 field populations (19 of *T. urticae* and six of *B. tabaci*) were collected between 2023 and 2025 and tested using single‐dose bioassays and droplet digital PCR (ddPCR) for target‐site resistance mutations.

**RESULTS:**

The results revealed significant variability in susceptibility to multiple acaricides and insecticides. Several *T. urticae* populations displaying reduced sensitivity to abamectin, hexythiazox, and fenpyroximate. Resistance mutations such as I321T (*GluCl3*), I1017F (*CHS1*), F1538I (*vgsc*), and G126S (*cytb*) were detected simultaneously at high frequencies in several *T. urticae* populations, indicating multi‐resistant phenotypes. A widespread occurrence of F331W (*ace1*), L925I/T929V (*vgsc*), and A2083V (*acc*) mutations were recorded in *B. tabaci*, showing the dynamics of resistance to organophosphates, pyrethroids, and ketoenols, respectively.

**CONCLUSION:**

Our findings underscore the ongoing evolution of resistance in these pests and highlight the need for integrated management strategies that include regular resistance monitoring and judicious pesticide use. This study provides resistance surveillance data to guide pest control decisions and promote sustainable Integrated Pest Management (IPM) in Greece. © 2025 The Author(s). *Pest Management Science* published by John Wiley & Sons Ltd on behalf of Society of Chemical Industry.

## Graphical abstract

Greek populations of *T. urticae* and *B. tabaci* exhibit evolving multi‐resistance, confirmed by bioassays and molecular markers, highlighting the urgent need for evidence‐based resistance monitoring in IPM.

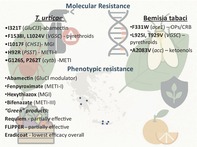

## 1 INTRODUCTION

The emergence and rapid spread of pesticide resistance in agricultural pests is a major threat to crop protection and food production. Resistance has been recorded in many arthropod species around the world and is often linked to the repeated use of insecticides. Two of the most important pests globally, the two‐spotted spider mite (*Tetranychus urticae*) and the silverleaf whitefly (*Bemisia tabaci*), are especially known for their ability to develop resistance to several groups of pesticides. According to the Arthropod Pesticide Resistance Database,^1^ there are currently 558 reported cases of resistance for *T. urticae* and 898 for *B. tabaci*.

In Greece, these pests are serious threats for many vegetable, fruit, and ornamental crops, both in greenhouses and in open fields. Integrated Pest Management (IPM) strategies are increasingly promoted as a holistic approach to pest control, encouraging the use of biological control agents, crop rotation, resistant cultivars, and environmentally friendly alternatives. Among these, the use of ‘green chemicals’ or biorational pesticides—products derived from natural sources such as plant extracts, microbial metabolites, or naturally occurring fatty acids—has gained ground in recent years. Although evidence is still accumulating, several categories of ‘green chemicals’ or biorational pesticides—such as botanical extracts, essential oils, microbial metabolites, and naturally occurring fatty acids—are generally regarded as having comparatively lower non‐target toxicity and potentially reduced resistance risks due to their complex mixtures of bioactive constituents. For these reasons, their use is increasingly promoted within IPM frameworks.^2^, ^3^, ^4^, ^5^ Nevertheless, chemical control remains the main method currently used in the field, particularly against *T. urticae* and *B. tabaci*.

The diversity and intensity of these applications contribute to strong selection pressure and accelerated resistance evolution. Several pesticide groups with different Modes of Action (MoA) are applied, including neonicotinoids, sulfoximines, and butenolides (which act on nicotinic acetylcholine receptors, *nAChRs*), abamectin (targeting the glutamate‐gated chloride channels, *GluCls*), pyriproxyfen (a juvenile hormone analog), hexythiazox, a mite growth inhibitor (MGI) targeting the chitin synthase *CHS1*, mitochondrial electron transport inhibitors (METI) like pyridaben, fenpyroximate, and acequinocyl, and diamides (affecting the ryanodine receptor, *RyR*). Due to their frequent use, resistance has been developed in many field populations of *T. urticae* and *B. tabaci*.^6^, ^7^, ^8^, ^9^, ^10^, ^11^, ^12^, ^13^, ^14^, ^15^, ^16^, ^17^, ^18^ One of the main mechanisms of resistance in these pests is the presence of mutations in the target sites of pesticides.

In *T. urticae*, several known mutations have been associated with resistance: I1017F in the *CHS1* gene (for MGIs like hexythiazox and etoxazole), F1538I and L1024V in the voltage‐gated sodium channel (*vgsc*) gene (linked with pyrethroid resistance), G126S, H92R, P262T and S141F in the mitochondrial cytochrome b gene (for resistance to METI‐I and METI‐III acaricides), and G314D, G326E, and I321T in the *GluCl* genes (for abamectin resistance).^6^, ^19^, ^20^, ^21^, ^22^

In *B. tabaci*, several resistance mutations have also been reported. These include the F331W mutation in the *ace1* gene (linked to resistance to organophosphates and carbamates),^23^ the L925I and T929V mutations in the *vgsc* gene (for pyrethroid resistance),^16^, ^24^ and the A2083V mutation in the *acc* gene (associated with resistance to ketoenols like spiromesifen).^25^ In addition, the A387G mutation in the cytochrome P450 gene *CYP6CM1* has been associated with neonicotinoid resistance (Pym *et al*., 2023), along with recently identified mutations in the β1 subunit of the nicotinic acetylcholine receptor (*nAChRβ1*) gene.^26^ To improve monitoring and resistance detection, droplet digital PCR (ddPCR) was recently developed as a highly sensitive molecular method to monitor the presence and frequency of resistance mutations with high accuracy and sensitivity,^10^, ^11^ in pooled insect and mite DNA samples.

In this study, we present bioassay and molecular monitoring resistance data for *T. urticae* and *B. tabaci* field populations collected from major crops and agricultural regions of Greece, collected between 2023 and 2025. Novel molecular assays were developed and applied for resistance mutations such as A387G in *CYP6CM1* and A58T in *BTβ1*. The results are discussed in the light of evidence‐based insecticide resistance management (IRM) and integrated pest management (IPM) strategies.

## 2 MATERIALS AND METHODS

### 2.1 *Tetranychus urticae* and *Bemisia tabaci* field samples

A total of 19 field populations of *T. urticae* (Tu1–Tu19) and six populations of *B. tabaci* (Bt1–Bt6) were collected from different areas of Greece between September 2023 and January 2025 (Supplementary Table S1). Sampling was carried out from infested leaves of various host plants mainly vegetables and citrus, collected from greenhouse and open field crops. Collection areas included major agricultural zones in Southern Greece such as the Peloponnese (Achaea, Argolida, Messinia) and Crete (Heraklion and Lasithi prefectures).

Four *T. urticae* populations were collected from citrus crops (lemon, orange, tangerine), 14 populations were collected from vegetables (cucumber, zucchini, tomato, eggplant, melon, watermelon, pepper), and one population in a garden growing rose. For several populations, pesticide application history was known and included a wide range of compounds. In particular, Tu5 (from pepper/eggplant in Tympaki) had a history of treatments with spinetoram, spinosad, metaflumizone, formetanate, and acrinathrin. Tu9 (from eggplant in Messinia) was treated with pyriproxyfen, acetamiprid and flonicamid, while Tu11 (from watermelon in the same area) had been treated with abamectin. Tu12 (from melon) was exposed to acetamiprid and chlorantraniliprole, Tu16 (zucchini) to pyriproxyfen, and Tu18 (zucchini) to hexythiazox, acequinocyl and fatty acid potassium salts. For the rest of the populations, either no chemical treatments had been applied (Tu1, Tu4, Tu6, Tu7, Tu8, Tu17, Tu19) or the pesticide history was unknown (Tu2, Tu3, Tu10, Tu13, Tu14, Tu15).

The *B. tabaci* populations were collected mainly from solanaceous and cucurbit crops such as eggplant, tomato, pepper, cucumber and melon. In this case, pesticide use was recorded for several samples. Bt1 (eggplant, Tympaki) had been exposed to flonicamid, acetamiprid, pyridaben, pyriproxyfen, and sulfoxaflor; Bt2 (tomato, Tympaki) to azadirachtin, acetamiprid, and pyriproxyfen; Bt5 to cyantraniliprole, sulfoxaflor, pyriproxyfen, pyridaben and deltamethrin. Although the Bt6 (eggplant, Tympaki) field was known to be untreated during the sampling period, it had a heavy history of chemical applications. In the remaining cases (Bt3, Bt4), the treatment history was unknown.

### 2.2 Toxicity assays

Mortality assessment was performed for *T. urticae* with four acaricides/insecticides of different MoA, abamectin (Vertimec 18EC, MoA: *GluCl* allosteric modulator/IRAC code 6), bifenazate (Floramite 240SC, MoA: METI‐III/ IRAC code 20D), fenpyroximate (Kento 5.12SC, MoA: METI‐I/ IRAC code 21A) and hexythiazox (Nissorun 25SC, MoA: MGI/IRAC code 10A) using diagnostic recommended field doses (RFD) of 18, 60, 102.4 and 75 mg*・*L^−1^, respectively. In addition, toxicity assays at the RFD were conducted with three ‘green’, non‐conventional chemistry formulations, namely Requiem, Eradicoat, and FLiPPER. Requiem is a botanical extract of the plant *Chenopodium ambrosioides* and contains a variety of terpenoids as active ingredients. The active ingredient of Eradicoat is maltodextrin, that is derived from decomposed potato starch. FLiPPER is a natural extract of olive oil and contains potassium salts, composed of unsaturated carboxylic acids. In all cases adulticidal bioassays were conducted with 25, 2–3 days old, adult female mites which were transferred on the upper side of 9 cm^2^ square‐cut kidney bean leaf discs on wet cotton wool. The plates were sprayed with 1 mL of spray fluid at 1 bar pressure with a Potter Spray Tower (Burkard Scientific, UK) to obtain a homogenous spray film. The toxic effects of hexythiazox were assessed with egg bioassays, by having 20 adult females laying eggs on a leaf disc for 5 h, as previously described.^27^ After spray treatment, leaf discs were placed at 25 ± 0.5 °C, 60% RH and 16:8 h (light: dark) photoperiod. Three replicates were sprayed per dose and, as a control, deionized water was used. Mortality was assessed after 72 h for abamectin, bifenazate, fenpyroximate, Requiem, Eradicoat and FLiPPER. Adult mites were considered alive if they could walk twice the distance of their body size after being prodded with a fine hairbrush. Mites treated with hexythiazox were considered unaffected, if they displayed the same developmental stage as the water treated control at the time of scoring. The percentage mortality resulting from exposure to each insecticide was calculated after being corrected for control mortality. The percentage mortality for each insecticide was corrected using Schneider‐Orelli's formula, *i.e*., Corrected % mortality = [(mortality % in treatment—mortality % in control) / (100—mortality % in control)] x 100.

Toxicity assays were performed only for *T. urticae*. Despite repeated sampling efforts, the number of available *B. tabaci* adults was insufficient to support replicated bioassays with adequate statistical power. Therefore, resistance in *B. tabaci* was assessed exclusively through ddPCR‐based detection of target‐site mutations, a strategy previously shown to strongly correlate with resistance phenotype in Greek field populations (Mavridis *et al*., 2022).

### 2.3 Genomic DNA isolation and quantification

Genomic DNA extractions were performed on pools of adult mites, following the CTAB method as previously described.^28^ The concentrations of extracted gDNAs were determined with the Qubit™ dsDNA BR Assay (Invitrogen, Carlsbad, CA) on a Qubit fluorometer 2.0 (Invitrogen, Carlsbad, CA), to obtain measurements specific for double stranded DNA (dsDNA). The mean ± SE dsDNA concentration for *T. urticae* pools was 12.2 ± 3.76 (range: 4.6–25.0 ng・μL^−1^), while for the *B. tabaci* pools 77.9 ± 19.85 (range: 11.0–140.0 ng・μL^−1^).

### 2.4 Droplet digital PCR (ddPCR) assays

Droplet digital PCR (ddPCR) was performed using the QX200 Droplet Digital PCR System (Bio‐Rad, Hercules, CA, USA), as previously described^10^, ^11^ Each 20 μL reaction contained 1× ddPCR Supermix for Probes (no dUTP) (Bio‐Rad), 5 U of EcoRI‐HF® restriction enzyme (New England Biolabs), 5 ng of genomic DNA, and primers and probes specific to each mutation. Probes were labeled with FAM for mutant alleles and HEX for wild‐type alleles.

Droplets were generated with the QX200 droplet generator and thermocycled under the following conditions: 95 °C for 10 min, followed by 50 cycles of 94 °C for 30 s and 54–60 °C for 1 min, and a final step at 98 °C for 10 min. After amplification, droplets were read on the QX200 droplet reader, and data were analyzed using QuantaSoft™ Analysis Pro Software (v1.0.596).

The limit of detection (LoD) was determined using synthetic gBlock™ controls and reached 0.1–0.2%, depending on the assay. This sensitivity allows for reliable detection of low‐frequency resistance alleles in pooled samples. Details on assay characteristics, including primers and probe sequences for previously published mutations, are available in Mavridis *et al*.^10^, ^11^ In addition, novel TaqMan ddPCR assays were developed for the detection of the A387G mutation in the *CYP6CM1* gene and the A58T mutation in the *BTβ1* gene.

For A387G, the primers used were: Bt_387_F: GTCTGTTCAGAGACGCTGCG and Bt_387_R: GTGCACGTCCGCACCAA and Probes: Bt_387_Pwt (wild‐type): HEX‐CTGTACCCGGCGTC‐MGB and Bt_387_Pmut (mutant): FAM‐CGACCCCGGGTAC‐MGB. For A58T, the primers were: Bt_58F: GCAGAACATCACTGAGAAGGTTGA and Bt_58R: TTTGTAAGACTCCGTTTTTATTTCGA and Probes: Bt_58_Pwt (wild‐type): HEX‐TTCGGGCTGGCTTT‐MGB and Bt_58_Pmut (mutant): FAM‐ATTCGGGCTGACTT‐MGB.

### 2.5 Statistical analyses

The LoD for each ddPCR assay was defined as the lowest mutant allelic frequency (MAF) that could be reliably detected with accuracy calculated according to.^29^ The MAF was calculated as: %MAF = [Mutant copies / (Mutant + Wild‐type copies)] × 100.

## 3 RESULTS

### 3.1 Toxicity assays

Toxicity assays were conducted on 12 field populations of *T. urticae* to evaluate their response to four acaricides with different MoA: abamectin (*GluCl* allosteric modulator, IRAC MoA code 6), bifenazate (METI‐III, IRAC MoA code 20D), fenpyroximate (METI‐I, IRAC MoA code 21A), and hexythiazox (MGI, IRAC MoA code 10A). The majority of the populations were collected from vegetables (eight out of 12) while the remaining four populations (Tu1‐Tu4) originated from citrus orchards. The results revealed strong variability in acaricide efficacy among populations (Table 1).

**Table 1:** Toxicity assays of four acaricides for 12 *T. urticae* field populations from Greece

Region—Host plant	Acaricide class (active ingredient)	*GluCl* allosteric modulators (IRAC MoA code 6) (Abamectin)	METI‐III (IRAC MoA code 20D) (Bifenazate)	METI‐I (IRAC MoA code 21A) (Fenpyroximate)	MGI (IRAC MoA code 10A) (Hexythiazox)
	Population	Corrected Mortality at the recommended field dose (%Mortality ± SE)			
Peloponnese—Citrus	Tu1	98.47 ± 1.53	100.00 ± 0.00	92.22 ± 4.07	99.35 ± 0.65
	Tu2	100.00 ± 0.00	100.00 ± 0.00	95.89 ± 2.26	99.29 ± 0.71
	Tu3	100.00 ± 0.00	96.33 ± 1.89	87.97 ± 5.12	100.00 ± 0.00
	Tu4	98.51 ± 1.49	100.00 ± 0.00	98.63 ± 1.37	100.00 ± 0.00
Crete—Vegetables	Tu5	36.99 ± 1.18	63.46 ± 9.67	9.46 ± 1.35	14.44 ± 2.09
	Tu6	100.00 ± 0.00	100.00 ± 0.00	100.00 ± 0.00	100.00 ± 0.00
	Tu13	100.00 ± 0.00	100.00 ± 0.00	100.00 ± 0.00	100.00 ± 0.00
	Tu14	100.00 ± 0.00	100.00 ± 0.00	100.00 ± 0.00	100.00 ± 0.00
Peloponnese—Vegetables	Tu9	24.6 ± 0.99	100.00 ± 0.00	28.83 ± 1.66	0.33 ± 0.58
	Tu10	13.10 ± 1.56	100.00 ± 0.00	19.66 ± 1.07	0.28 ± 1.38
	Tu11	100.00 ± 0.00	100.00 ± 0.00	100.00 ± 0.00	100.00 ± 0.00
	Tu12	100.00 ± 0.00	100.00 ± 0.00	100.00 ± 0.00	100.00 ± 0.00

Most tested populations (Tu1–Tu4, Tu6, Tu11–Tu14) exhibited high levels of susceptibility to all four compounds, with corrected mortality values ranging from 87.57% to 100%. However, three populations (Tu5, Tu9, and Tu10) showed reduced sensitivity to multiple acaricides. Tu5 showed especially low mortality to abamectin (36.99%), fenpyroximate (9.46%), and hexythiazox (14.44%) and low to moderate mortality to bifenazate (63.46%), suggesting resistance to several chemical groups. Similarly, Tu9 and Tu10 showed very low mortality to hexythiazox (0.33% and 0.28%), abamectin (24.6% and 13.10%) and fenpyroximate (28.83% and 19.66%), indicating multiple resistance traits.

Among the tested products, bifenazate was the most effective acaricide across the evaluated populations, consistently achieving 100% mortality in all except one (Tu5), where a moderate yet significant reduction in efficacy was observed (63.46%).

Five *T. urticae* populations were also tested against three alternative ‘green’ products: Requiem, Eradicoat and FLiPPER (Table 2). Population Tu3 derived from citrus while the remaining four populations (Tu5, Tu9, Tu10 and Tu14) are from vegetables. In general FLiPPER and Requiem were the most effective products, producing >70% mortality in four out of five populations. Population Tu10 showed high susceptibility to all three products, with mortality rates above 90%. Tu9 and Tu14 both showed high mortality to Requiem and FLiPPER (100%), but their response to Eradicoat was lower, with Tu9 showing reduced mortality (43.47%) and Tu14 showing a moderate response (71.93%). Tu3 showed moderate mortality to Requiem (74.64%) and FLiPPER (70.68%), but a low response to Eradicoat (23.58%). Tu5 was the least responsive, with very low mortality to Eradicoat (6.44%) and low response to Requiem (42.73%) and FLiPPER (39.27%).

**Table 2:** Toxicity assays of three green pesticides for 5 *T. urticae* field populations from Greece

Green pesticide	Requiem	Eradicoat	FLiPPER
Population	Corrected Mortality at the recommended field dose (%Mortality ± SE)		
Tu3	74.64 ± 2.58	23.58 ± 7.25	70.68 ± 4.67
Tu5	42.73 ± 15.94	6.44 ± 5.41	39.27 ± 8.90
Tu9	100.00 ± 0.00	43.47 ± 2.14	100.00 ± 0.00
Tu10	91.97 ± 1.74	95.83 ± 1.62	100.00 ± 0.00
Tu14	100.00 ± 0.00	71.93 ± 1.62	100.00 ± 0.00

### 3.2 Detection of resistance mutations in *T. urticae* field populations

ddPCR analysis was used to quantify the frequency of resistance‐associated target‐site mutations in 17 *T. urticae* field populations. The results revealed substantial variability in resistance allele frequencies among populations and across different pesticides (Table 3).

**Table 3:** Mutant allele frequencies measured by ddPCR in pools of field *T. urticae* populations (*n* = 50 individuals per pool)

Region—Host plant	Acaricide class (active ingredient)	*GluCl* allosteric modulators—IRAC MoA code 6 (Abamectin)			Sodium channel modulators—IRAC MoA code 3A (Pyrethroids)		MGI—IRAC MoA code 10 (Etoxazole/Hexythiazox)	METI‐I—IRAC MoA code 21A (Pyridaben)	METI‐III—IRAC MoA code 20D (Bifenazate)		
	Mutation	G314D	G326E	I321T	F1538	L1024V	I1017F	H92R	G126S	S141F	P262T
	Gene	*GluCl1*	*GluCl3*		*vgsc*		*CHS1*	*PSST*	*cytb*		
	Population	%MAF (Mutant Allelic Frequency)									
Peloponnese—Citrus	Tu1	0.00	0.00	0.00	0.00	0.92	1.89	0.00	0.00	0.00	0.00
	Τu2	0.00	0.00	0.39	0.00	0.74	0.40	0.00	0.41	0.00	0.00
	Tu3	0.00	0.00	0.00	0.00	0.00	0.00	0.00	0.00	0.00	0.00
	Tu4	0.00	0.00	0.00	0.00	0.00	0.00	0.00	0.00	0.00	0.00
Crete—Vegetables	Tu5	0.00	0.00	54.45	100.00	0.00	100.00	0.74	100.00	0.00	0.00
	Tu7	0.00	0.00	30.04	0.00	0.00	0.00	0.00	0.00	0.00	0.00
	Tu13	0.00	0.00	0.00	4.64	0.00	0.00	0.00	0.00	0.00	0.00
	Tu14	0.00	0.00	0.00	0.00	0.00	0.00	0.00	0.00	0.00	0.00
	Tu15	0.00	0.00	0.00	31.41	0.00	0.00	0.00	0.00	0.00	0.00
	Tu16	0.00	0.00	69.04	92.33	0.00	91.17	0.00	92.79	0.00	0.00
	Tu17	0.00	0.00	0.00	0.00	0.00	0.00	0.00	0.00	0.00	0.00
	Tu18	0.00	0.00	10.07	0.00	0.00	0.00	0.00	0.00	0.00	0.00
	Tu19	0.00	0.00	N/A	42.71	0.00	100.00	47.77	100.00	0.00	42.89
Peloponnese—Vegetables	Tu9	0.00	0.00	100.00	100.00	0.00	100.00	0.00	100.00	0.00	0.00
	Tu10	0.00	0.00	100.00	0.00	0.00	100.00	0.00	100.00	0.00	0.00
	Tu12	0.00	0.00	0.00	100.00	0.00	0.00	0.00	0.00	0.00	0.00
Crete—Other	Tu8	0.00	0.00	16.10	1.65	0.00	0.00	0.00	0.00	0.00	0.00

Mutations associated with resistance to *GluCl* allosteric modulators (abamectin), including G314D and G326E in *GluCl1* and *GluCl3* respectively, were absent in all populations (0% MAF). The I321T mutation in *GluCl3*, also linked to abamectin resistance, was detected in eight populations, in fixation (100%) in two populations (Tu9, Tu10), in high frequencies in Tu5 (54.45%), Tu16 (69.04%), and lower levels in Tu7 (30.04%), Tu8 (16.10%), Tu18 (10.07%), and Tu2 (0.39%).

For sodium channel modulators (pyrethroids), F1538I was widely present (8 populations), being fixed or nearly fixed in Tu5 (100%), Tu9 (100%), Tu12 (100%), and Tu16 (92.33%). Intermediate or lower frequencies were observed in Tu15 (31.41%), Tu19 (42.71%), Tu13 (4.64%), and Tu8 (1.65%). In contrast, L1024V was only detected at extremely low frequency in Tu1 (0.92%) and Tu2 (0.74%) both originating from citrus.

The I1017F mutation in *CHS1*, conferring resistance to mite growth inhibitors (MGIs) such as etoxazole and hexythiazox, was highly prevalent or fixed in five populations (Tu5, Tu9, Tu10, Tu16 and Tu19), and present at very low levels in Tu1 (1.89%) and Tu2 (0.40%).

Regarding mitochondrial electron transport inhibitors (METIs), H92R (associated with METI‐I resistance) was detected at moderate frequency in populations Tu19 (47.77%) and at extremely low frequency in Tu5 (0.74%). G126S was highly prevalent or fixed in Tu5, Tu9, Tu10, Tu16, and Tu19 while found at low levels in Tu2 (0.41%). The P262T mutation was detected only in Tu19 (42.89%), while S141F was not detected in any population.

No resistance mutations were detected in four populations (Tu3, Tu4, Tu14 and Tu17) suggesting that these populations are fully susceptible based on the tested markers. On the other hand, Tu5, Tu9, Tu10, Tu16, and Tu19 harbored multiple high‐frequency resistance mutations, indicating multi‐resistant phenotypes.

### 3.3 Detection of resistance mutations in *B. tabaci* field populations

Six *B. tabaci* field populations (Bt1–Bt6) were screened using ddPCR for key target‐site resistance mutations associated with multiple insecticide classes (Table 4).

**Table 4:** Mutant allele frequencies measured by ddPCR in pools of *B. tabaci* field populations

Insecticide class (active ingredient)	AChE inhibitors—IRAC MoA code 1 (OPs, carbamates)	Sodium channel modulators—IRAC MoA code 3A (Pyrethroids)		Lipid biosynthesis inhibitors targeting *acc*—IRAC MoA code 23 (tetronic & tetramic acid derivatives /ketoenols)
Mutation	F331W	L925I	T929V	A2083V
Gene	*ace1*	*vgsc*		*acc*
Population	%MAF (Mutant Allelic Frequency)			
Bt1	92.44	34.36	58.24	88.41
Bt2	89.64	35.72	56.48	89.82
Bt3	21.48	2.90	10.73	1.39
Bt4	95.56	31.65	51.83	71.43
Bt5	89.30	37.49	57.94	78.12
Bt6	92.11	44.74	45.29	92.74

The F331W mutation in the *ace1* gene, which confers resistance to acetylcholinesterase inhibitors (organophosphates and carbamates), was detected at high frequencies in all populations except Bt3 (21.48%), ranging from 89.30% in Bt5 to 95.56% in Bt4. For pyrethroid resistance, both L925I and T929V mutations in the *vgsc* gene were consistently detected. The combined MAFs for these two mutations ranged from low in Bt3 (L925I: 2.90%, T929V: 10.73%) to high levels in Bt6 (L925I: 44.74%, T929V: 45.29%). The A2083V mutation in the *acc* gene, associated with resistance to lipid biosynthesis inhibitors (tetronic and tetramic acid derivatives), showed strong variation. It was found to have a very high frequency in five out of six populations (Bt1, Bt2, Bt4, Bt5 and Bt6) ranging from 71.43% to 92.74% while detected at extremely low frequency in population Bt3 (1.39%).

Among the tested populations, Bt6 appeared as the most resistant, showing the highest MAFs across all tested classes: F331W (92.11%), L925I/T929V (44.74/45.29%), and A2083V (92.74%), indicating multiple target‐site resistance. In contrast, Bt3 exhibited the lowest MAFs, F331W (21.48%), L925I/T929V (2.90/10.73%), and A2083V (1.39%), suggesting a more susceptible profile.

None of the tested neonicotinoid resistance mutations‐A387G in the *CYP6CM1* gene and A58T in the *BTβ1* gene‐were detected in any of the *B. tabaci* populations analyzed.

## 4 DISCUSSION

This study examined field populations of *T. urticae* and *B. tabaci* from key agricultural regions in Southern Greece, encompassing both mainland areas and the island of Crete. These regions are marked by intensive fruit and vegetable cultivation‐under both open‐field and greenhouse conditions‐where pest control predominantly depends on repeated pesticide applications. Such high‐input practices impose strong selection pressure, accelerating the emergence and spread of resistance in local pest populations.

Our study confirms the presence of widespread resistance in both species in Greece, with strong associations between bioassay results and key resistance mutations. The toxicity data for four acaricides (abamectin, bifenazate, fenpyroximate, and hexythiazox) demonstrated a wide range of responses among *T. urticae* populations. Among the 12 populations tested, four originated from citrus orchards and eight from vegetable crops. Citrus populations were almost fully susceptible to all tested acaricides, whereas populations from vegetables showed varying responses at the recommended field dose. This pattern reflects the much higher and more frequent pesticide inputs typically applied in intensive vegetable systems compared to citrus orchards, where fewer chemical interventions are carried out. Three populations from vegetables, collected in Messinia (Peloponnese) and Tympaki (Crete), displayed low sensitivity to three or all four tested acaricides, indicating multi‐resistant phenotypes. In our previous study,^11^ more than half of the tested populations were found to be multi‐resistant to the majority of acaricides. This difference may be attributed to the fact that those populations were collected from Crete 5 years ago, from heavily treated greenhouse systems growing vegetables and ornamentals.

The variable efficacy of Requiem, Eradicoat, and FLiPPER across *T. urticae* populations highlights the complex interplay between population‐specific resistance mechanisms and the unique modes of action of these green products. Their ability to control some multi‐resistant populations (e.g., Tu9 and particularly Tu10) but not others (e.g., Tu5) indicates that resistance mechanisms effective against conventional acaricides do not necessarily confer cross‐resistance to these materials, which rely largely on physical or multi‐site physiological effects rather than single molecular targets. The unexpected insensitivity of the highly susceptible Tu3 population further supports the idea that factors such as cuticular properties, detoxification capacity, or penetration‐related traits drive responses to these products independently of traditional target‐site resistance. From an IPM perspective, these characteristics make green products valuable complementary tools, suitable for rotation or mixture strategies aimed at lowering selection pressure on conventional acaricides and maintaining long‐term control efficacy. However, their inconsistent performance across populations underscores the need for local resistance monitoring and population‐specific decision‐making to ensure their effective and sustainable integration into resistance management programs.

A panel of 10 target‐site resistance mutations, previously developed and applied,^11^ was used to detect resistance in *T. urticae*. These assays allowed us to identify multiple mutations associated with resistance, some of which have also been linked to phenotypic resistance and provided useful comparisons with previous findings.

The distribution of resistance mutations varied across regions (Crete and Peloponnese) and host plants (vegetables and citrus) (Table 3). Citrus‐derived populations, collected exclusively from the Peloponnese, exhibited resistance alleles at absent or extremely low frequencies, whereas vegetable‐associated populations from both regions showed moderate to high frequencies of resistance mutations. This differentiation is consistent with established evidence that resistance evolution in *T. urticae* is driven largely by the intensity of pesticide selection pressure, which is typically much higher in vegetable systems that receive frequent, multi‐chemical applications. By contrast, citrus orchards generally rely on fewer pesticide treatments and more integrated control approaches, resulting in weaker selection for resistance. Host‐plant effects may further contribute, as many vegetable crops support higher mite fecundity and faster population turnover—conditions that promote rapid resistance development—while citrus tends to support lower population growth rates. Together, these agronomic and ecological differences likely explain the contrasting resistance patterns observed between citrus and vegetable populations.

Mutations in *GluCl1* (G314D) and *GluCl3* (G326E and I321T) have been strongly associated with abamectin resistance.^6^, ^12^, ^30^ Among the three, only I321T was detected in our study, in eight populations with varying frequencies, whereas G314D and G326E were completely absent. In populations Tu5, Tu9, and Tu10—where both toxicity and molecular data were available—there was a clear correlation between low mortality and high mutation frequency. Compared to our previous study,^11^ where G326E alone or in combination with G314D was detected in four out of six populations and associated with low mortality at RFD (as low as 6%), the present data show a shift toward the predominance of the I321T mutation. According to Xue *et al*. (2020),^30^ the I321T mutation was found exclusively in red‐morph *T. urticae* strains exhibiting abamectin resistance, and similarly, all populations harboring I321T in the present study were also of the red morph, suggesting a consistent association. It is also possible that additional mutations in *GluCl3* (V327G and L329F^30^) or the recently reported insertions in *GluCl2*,^31^ which were not investigated here, may be present and contribute to the observed resistance phenotypes.

F1538I and L1024V are known pyrethroid resistance mutations.^18^, ^32^ L1024V was detected at very low frequency in only two citrus populations (<1%), while F1538I was more frequent, found in nearly half of the tested populations and at high frequency in several cases. This prevalence is consistent with our previous findings.^11^ Although pyrethroids are not registered against *T. urticae* in citrus or vegetables, their use against other insect pests may explain the selection pressure and the observed high frequencies in populations from vegetables.

The I1017F mutation in *CHS1* was found at high frequency in populations with low hexythiazox mortality, confirming its strong link to MGI resistance. It was found fixed in three populations from vegetables which showed poor response to hexythiazox. These results agree with our previous study,^11^ where this mutation was detected at high frequency in half of the populations and was also correlated with resistance to etoxazole.

H92R, a mutation in *PSST* associated with resistance to METI‐I acaricides (e.g., pyridaben, fenpyroximate), was detected in only two populations—one at moderate (48%) and one at very low frequency (<1%). Interestingly, toxicity data showed low mortality to fenpyroximate in three populations (Tu5, Tu9, Tu10), although the H92R mutation was not present. Previously,^11^ H92R was frequently found in Crete populations, though it showed poor association with pyridaben toxicity indicating the possible participation of other resistance mechanisms such as metabolic resistance.

For bifenazate resistance, three *cytb* mutations (G126S, S141F, and P262T) were examined. G126S was found at high or fixed frequency in several populations, while P262T was detected in only one case, in combination with G126S. In our previous study (Mavridis *et al*., 2022b), only G126S was detected, consistent with the current findings—with one exception where P262T was also identified. To our knowledge, this is the first detection of P262T in Greece and may reflect evolving resistance to bifenazate. In the three populations for which both phenotypic and genotypic data were available (Tu5, Tu9, Tu10), no direct association between mutation frequency and bifenazate mortality was evident, as only Tu5 displayed reduced susceptibility. Given that G126S alone has been demonstrated not to confer resistance,^33^ the high frequency of this mutation in Tu5 likely interacts with additional *cytb* mutations previously implicated in bifenazate resistance but not examined here^34^, ^35^ to account for the observed phenotype.

Taken together, these findings illustrate that, although target‐site mutations provide valuable insight into resistance patterns, they do not always fully explain the phenotypes observed in field populations such as Tu5. This reflects a broader limitation of the present study: only a subset of known cytb and other target‐site mutations were examined, and metabolic resistance mechanisms—known to play a major role in *T. urticae*—were not assessed. A more comprehensive approach integrating additional markers and transcriptomic or biochemical analyses will be essential to elucidate the complete resistance basis of multi‐resistant populations.

In *B. tabaci*, most populations exhibited multiple target‐site resistance mutations, including F331W in *ace1*, L925I and T929V in *vgsc*, and A2083V in *acc*. The F331W mutation, previously reported as fixed in Crete populations,^18^ remained at very high frequencies (>89%) in all samples except Bt3 (21.48%).

Likewise, both L925I and T929V occurred at moderate to high frequencies across nearly all populations, with Bt3 again representing the only notable exception. In contrast to our earlier survey,^10^ in which L925I was more prevalent than T929V, the present data suggest a slight shift toward higher T929V frequencies, possibly reflecting competitive or mutually exclusive interactions between the two alleles. High frequencies of kdr mutations have also been documented in previous studies from Greece and other Mediterranean regions^36^, ^37^ supporting the view that pyrethroid selection pressure has been historically intense in these agroecosystems.

The persistence and widespread distribution of these mutations—often at near fixation levels and even in the presence of multiple resistance alleles—suggests that they impose minimal fitness costs, consistent with previous findings for kdr and *ace1* mutations in *B. tabaci* and other hemipteran pests.

The A2083V mutation, associated with ketoenol resistance, was found at high to very high frequencies (>71%) in five out of six populations, and at very low frequency in Bt3. In our previous work, this mutation was detected in 8 of 11 populations, but usually at lower levels (>70% in only three populations). Although we did not perform spiromesifen bioassays in the current study, high resistance levels against the compound have been documented in *B. tabaci* populations from Greece^38^ and our earlier findings^10^ support a strong genotype–phenotype link for A2083V.

Neonicotinoid resistance in *B. tabaci* has been functionally associated with the overexpression of *CYP6CM1*, which enhances the metabolic detoxification of neonicotinoid molecules into less toxic derivatives.^39^, ^40^ In the present study, ddPCR assays were developed to target recently reported mutations in the *CYP6CM1* and *nAChRβ1* genes.^26^ However, neither mutation was detected in any of the populations examined. This absence may be attributable to the prohibition of neonicotinoids in open‐field crops, including vegetables, since 2018 in Greece, following European Union legislation. Nevertheless, the molecular diagnostics developed here represent a promising tool for future resistance monitoring programs.

## 5 CONCLUSION

This study reveals a complex and evolving resistance landscape in *T. urticae* and *B. tabaci* across major agricultural regions of Greece. Resistance patterns closely reflect cropping systems and pesticide usage intensity, with populations from vegetable crops in Crete and Messinia exhibiting higher levels of resistance compared to the more susceptible populations from citrus orchards. This highlights the critical role of local agronomic practices in shaping resistance dynamics and underscores the need for tailored pest management strategies across cultivation zones.

The frequent co‐occurrence of multiple target‐site mutations within the same populations points to widespread multi‐resistance and declining efficacy of commonly used pesticides. These findings reinforce the importance of integrating routine bioassays with molecular diagnostics to enable early detection and informed decision‐making in pest control. Resistance monitoring should be a cornerstone of IPM programs, especially in high‐input systems where resistance across several mode‐of‐action classes is already entrenched.

Looking ahead, the sustainable management of these pests will require the adoption of innovative tools such as biopesticides.

Combining conventional and emerging approaches within a coordinated, evidence‐based framework will be essential to preserve crop productivity and enhance agroecosystem resilience.

## CONFLICT OF INTEREST STATEMENT

The authors declare that they have no conflicts of interest related to this work.

## Supporting information


**Table S1.** Collection details of *Tetranychus urticae* (Tu1–Tu19) and *Bemisia tabaci* (Bt1–Bt6) field populations, including locality, host plant, and date.

## Data Availability

The data that support the findings of this study are available from the corresponding author upon reasonable request.
