# Supplementary material for: Evolving resistance patterns in Tetranychus urticae and Bemisia tabaci in Greece
Source: Pest Manag Sci. 2025 Dec 21;82(4):3544–51. doi: 10.1002/ps.70475 (PMC12976199; doi:10.1002/ps.70475)
Supplement: Supplementary file 1 — Table S1. Collection details of Tetranychus urticae (Tu1–Tu19) and Bemisia tabaci (Bt1–Bt6) field populations, including locality, host plant, and date. [file PS-82-3544-s001.docx]

**Supplementary Table S1**. Collection details of *Tetranychus urticae* (Tu1–Tu19) and *Bemisia tabaci* (Bt1–Bt6) field populations, including locality, host plant, and date.

| **Population** | **Locality** | **Host Plant** | **Collection Date** |
| --- | --- | --- | --- |
| **Tu1** | Trypia/Aigion, Achaea Prefecture | Lemon | 19 September 2023 |
| **Tu2** | Elaionas/Aigion, Achaea Prefecture | Lemon | 2 October 2023 |
| **Tu3** | Leukakia, Argolida Prefecture | Orange | 9 November 2023 |
| **Tu4** | Kalithea, Argolida Prefecture | Tangerine | 10 November 2023 |
| **Tu5** | Tympaki, Heraklion Prefecture | Pepper/Eggplant | 23 November 2023 |
| **Tu6** | Kalyviani, Heraklion Prefecture | Pepper | 1 February 2024 |
| **Tu7** | Ampelouzos, Heraklion Prefecture | Eggplant | 6 February 2024 |
| **Tu8** | Ampelouzos, Heraklion Prefecture | Rose | 6 February 2024 |
| **Tu9** | Terpsithea/Kyparissia, Messinia Prefecture | Eggplant | 1 June 2024 |
| **Tu10** | Faraklasda/Kyparissia, Messinia Prefecture | Cucumber | 1 June 2024 |
| **Tu11** | Agia Kyriaki/Kyparissia, Messinia Prefecture | Watermellon | 1 June 2024 |
| **Tu12** | Marathoupoli/Kyparissia, Messinia Prefecture | Mellon | 1 June 2024 |
| **Tu13** | Fourni, Lasithi Prefecture | Various Vegetables | 30 June 2024 |
| **Tu14** | Neapoli, Lasithi Prefecture | Various Vegetables | 30 June 2024 |
| **Tu15** | Kalessa, Heraklion Prefecture | Ζucchini | 22 July 2024 |
| **Tu16** | Tympaki, Heraklion Prefecture | Zucchini | 24 July 2024 |
| **Tu17** | Krousonas, Heraklion Prefecture | Tomato | 31 July 2024 |
| **Tu18** | Tympaki, Heraklion Prefecture | Ζucchini | 7 October 2024 |
| **Tu19** | Tympaki, Heraklion Prefecture | Εggplant | 21 January 2025 |
| **Bt1** | Tympaki, Heraklion Prefecture | Εggplant | 24 July 2024 |
| **Bt2** | Tympaki, Heraklion Prefecture | Tomato | 24 July 2024 |
| **Bt3** | Kalessa, Heraklion Prefecture | Cucumber | 29 August 2024 |
| **Bt4** | Moires, Heraklion Prefecture | Μelon | 9 July 2024 |
| **Bt5** | Tympaki, Heraklion Prefecture | Pepper | 21 January 2025 |
| **Bt6** | Tympaki, Heraklion Prefecture | Εggplant | 21 January 2025 |

*****Refers to the last season prior population collection
